# Buffalo Obstetrical Society

**Published:** 1885-03

**Authors:** 


### Buffalo Obstetrical Society.

This society, which has prosperously entered upon the second year of its existence, bids fair to be one of the most useful and important of the various medical organizations for professional improvement in our city. Its membership list includes some of the most prominent and experienced of our physicians, and its Transactions, which we publish in each issue of the Journal, afford much interesting matter, which our readers will find well worthy of careful perusal. At the last annual meeting of the society the following named officers were re-elected for the ensuing year: President, W. W. Potter, M. D.; Vice-President, R. L. Banta, M. D.; Secretary and Treasurer, William H. Thornton, M. D.

---

WE have noted, for some months, the frequent reports in medical journals of attempts at abortion practiced by women on themselves. In all these cases the instrument has been a hair-pin introduced into the uterus with the points down, thus preventing ready removal in case the instrument should slip from their grasp. It is only in these cases that the physician is called. Besides these there are catheters, knitting needles, wires and various other articles used. It only shows to what an alarming extent women resort to mechanical and dangerous means to prevent maternity.

---

E. S. GAILLARD, A. M., M. D., LL. D., editor of *Gaillard's Medical Journal*, died February 7th, at Ocean Beach, N. J. In 1866 he founded the *Richmond Medical Journal*, which, upon his removal to Louisville in 1868, was continued as the *Richmond and Louisville Medical Journal*. Subsequently he removed to New York where he established *Gaillard's Medical Journal*. This he conducted with great ability to the day of his death. We are informed that this publication will be continued by M. E. and E. W. Gaillard with an able corps of collaborators.

---

DR. PERCY KIDD recently contributed a notable paper to the Royal Medical and Chirurgical Society on "The Distribution of the Tubercle Bacillus." The paper and the extended discussion of it that followed its reading served to show a remarkable unanimity of opinion among physicians as to the presence of the tubercle bacillus in the products of phthisis, including the sputum, and little doubt was expressed as to its etiological significance. "What shall be done with this organism?" was variously answered.

---

THE London patient on whom Mr. Godlee operated and removed a gliomatous tumor from the brain successfully, progressed favorably for a few days, but afterwards died of cerebritis. Notwithstanding the fatal termination of the case, it demonstrated a great achievement in diagnosis and cerebral localization on the part of Dr. Hughes Bennett, and foreshadows a future of cerebral surgery with possibilities of great reward in relieving suffering and saving life.

---

Mr. T. Pridgin Teale, in a clinical lecture "On the Surgery of Scrofulous Glands," expresses the opinion that scrofulous glands, "even when not suppurating, are centres from which health-damaging and death-dealing material may be diffused throughout the human frame," and that these centres should be removed by enucleation and dissection, or by "scraping" by means of Lister's scraper.

---

Dr. Osler, in the last number of the *Archives of Medicine*, refers, in an article, to the model cattle market and *abattoir* of Berlin. Private slaughtering is strictly forbidden, and the carcass of every animal slaughtered is submitted to the critical eye of one of the inspectors, of whom there are no less than 141, who must be satisfied that the carcass is healthy before it is exposed for sale.

---

The vacancy in the University of Leipsic, caused by the death of Prof. Cohnheim, in August last, has been filled recently by the appointment of Prof. Birch-Hirschfeld, of Dresden.

---

Sir Joseph Lister, Bart., has been appointed, by the Emperor of Germany, a Knight of the Order Pour le Merite for Science and Art—an honor very rarely conferred.

---

The announcement for the Spring Course af Lectures in the Niagara Medical College is at hand. The term will begin April 1, 1885, and continue eight weeks.

---

The *Archives of Medicine*, hailing from Putman's, New York, has suspended. Such is the fate, also, of the *Index Medicus*.

